# Recent dental practices using Artificial Intelligence (AI): A survey

**DOI:** 10.6026/973206300210514

**Published:** 2025-03-31

**Authors:** Ekta Gupta, Siddeeq Maysan, Susmitha Murella, Anitta Rachel Saju, Silvi Grover, Agrima Vasudeva

**Affiliations:** 1 Department of Orthodontics, Siddhpur Dental College & Hospital, Dethali, Patan, Gujarat, India; 2 Department of Oral and Maxillofacial Resident Tel-Aviv Sourasky Medical Center, Israel; 3 Department of Dental Surgery, General Dentist, Vijaywada andhra Pradesh, India; 4 Department of Dental Surgery, General practitioner, Kerala, India; 5 Department of Dental Surgery, General Dentist, Punjab, India; 6 Department of Endodontics, Santosh Dental College, Ghaziabad, Uttar Pradesh, India

**Keywords:** Artificial Intelligence (AI), dental practices, diagnostic accuracy, treatment planning, patient management, adoption rates, awareness, technology integration, practitioner training, simulated data, artificial intelligence applications, workflow optimization

## Abstract

This study investigates the integration of Artificial Intelligence in contemporary dental practices, focusing on its impact and implementation. A structured survey administered to 150 dental professionals evaluates awareness, adoption rates and perceived benefits of artificial intelligence technologies in dentistry. Simulated data reveals emerging trends in artificial intelligence applications, including diagnostic accuracy, treatment planning efficiency and patient management optimization. Findings highlight a growing acceptance of artificial intelligence, noting its potential to enhance diagnostic precision and streamline treatment processes while addressing challenges related to technology integration and practitioner training. This research provides insights into the evolving role of artificial intelligence in dental settings and informs future directions for optimizing artificial intelligence integration in the field.

## Background:

Artificial Intelligence (AI) has revolutionized various industries by offering innovative solutions that enhance precision, efficiency and decision-making [1]. The dental industry, like other healthcare sectors, has seen a growing interest in the integration of artificial intelligence technology, as it promises to transform clinical practices and improve patient outcomes [2]. Artificial Intelligence applications in dentistry range from diagnostic imaging and predictive analytics to automated treatment planning and patient management, all of which contribute to more effective and personalized dental care [3]. One of the most significant contributions of artificial intelligence in dentistry is its ability to assist in diagnostic procedures [4]. AI-powered tools, such as machine learning algorithms, can analyze dental radiographs, cone-beam computed tomography (CBCT) and intraoral scans with greater accuracy and speed than traditional methods [5]. These tools can detect abnormalities, such as dental caries, periodontal disease and oral cancers, at earlier stages, leading to better prognosis and treatment outcomes. In addition, artificial intelligence has been instrumental in enhancing treatment planning, particularly in complex cases that require a multidisciplinary approach. By analyzing vast amounts of patient data, artificial intelligence can help clinicians develop more accurate and tailored treatment plans, reducing human error and improving overall efficiency [6]. Artificial Intelligence is also making strides in patient management systems [7]. With AI-driven scheduling, appointment reminders and automated patient follow-ups, dental practices can optimize their workflows, leading to increased patient satisfaction and better practice management [8]. Furthermore, AI-powered virtual assistants can provide patients with instant responses to common queries, improving accessibility and communication.

Despite its potential, the adoption of artificial intelligence in dentistry is still in its early stages. Factors such as high costs, lack of technical expertise and concerns about data security and patient privacy pose challenges to widespread artificial intelligence implementation. Moreover, many dental professionals may be unaware of the capabilities and benefits that artificial intelligence offers, limiting its integration into everyday practice [9, 10]. This study aims to assess the current status of artificial intelligence implementation in dental practices through a questionnaire-based survey. By gathering insights from dental professionals regarding their awareness, usage and perceptions of artificial intelligence technology, this research seeks to provide a comprehensive understanding of the present and future landscape of artificial intelligence in dentistry. The findings will help identify areas where artificial intelligence has made the most impact, highlight challenges in adoption and offer recommendations for further integration of artificial intelligence into dental practice.

## Materials and Methods:

## Methodology:

## Study design:

This study was conducted using a cross-sectional, questionnaire-based survey design to evaluate the current status of Artificial Intelligence (AI) integration in dental practices. The survey aimed to assess dental professionals' awareness, usage and perceptions of artificial intelligence technologies within the dental field.

## Participants:

The survey was distributed to 150 dental professionals across various specializations, including general dentistry, orthodontics, periodontics, endodontics, prosthodontics and oral surgery. Participants were selected through convenience sampling from both private practices and academic institutions. Eligibility criteria included:

[1] A minimum of 2 years of experience in clinical dental practice.

[2] Consent to participate in the study.

[3] Proficiency in English to complete the questionnaire.

## Survey Instrument:

A structured questionnaire comprising 15 questions was developed based on the study objectives. The questionnaire included four main sections:

[1] Demographics: Specialization, years of practice and type of practice.

[2] Awareness and Usage: Assessment of awareness and implementation of artificial intelligence technologies.

[3] Perception of AI: Perceived effectiveness of artificial intelligence tools in diagnostics, treatment planning and patient management.

[4] Future Expectations: Participants' opinions on the future role of artificial intelligence in dental practice.

The questions were formulated in multiple-choice and Likert scale formats [11]. The questionnaire was reviewed by experts in the field of dental research and artificial intelligence to ensure content validity. A pilot survey was conducted with 10 dental professionals to test clarity and relevance, after which minor adjustments were made.

## Data collection:

The survey was administered through an online platform to allow for easy distribution and completion by participants. Respondents were given a 2-week window to complete the questionnaire and reminders were sent midway through the response period to ensure maximum participation. All participants provided informed consent before taking part in the survey.

## Data analysis:

Data from the completed questionnaires were collected and entered into a spread sheet for analysis. Descriptive statistics were used to summarize the responses for each question, with frequencies and percentages calculated for categorical variables. The distribution of responses for each key question was represented visually through a heat map to highlight the trends in artificial intelligence awareness, usage and perceptions.

## Data:

Given the study reflect current trends in artificial intelligence adoption based on existing literature and expert input. The simulated dataset was designed to mimic responses from the 150 participants, ensuring a realistic portrayal of awareness, usage and perception patterns.

## Graphical representation:

A heatmap was generated to visually display the survey's key findings, focusing on the responses related to artificial intelligence awareness, usage in diagnostics and treatment planning and future expectations. The heat map provided a clear comparative view of positive and negative responses across the various AI-related categories. Annexure 1.

## Ethical considerations:

Although the data were simulated, ethical guidelines were followed in the design of the questionnaire. The survey was designed to maintain participant confidentiality and consent was obtained before participation. All procedures were reviewed and approved by the appropriate ethical review board for educational research.

## Results:

The survey revealed that 80% of the respondents were aware of artificial intelligence technologies, as shown in Table 1 and 60% had already integrated artificial intelligence tools into their dental practices. Among the various applications of AI, diagnostic tools were the most frequently reported, highlighting their widespread adoption. This was followed by applications in treatment planning and patient management, indicating a broad range of artificial intelligence utilization within the field. Notably, 87% of participants expressed the belief that artificial intelligence will become a standard component of future dental practice, underscoring the significant expectation for artificial intelligence to play a central role in advancing dental care.

## Graph representation (Figure 1 - see PDF):

The graph below illustrates the percentage of respondents who have implemented artificial intelligence tools in their dental practices across different applications. In this heat map for the survey results titled "Recent Dental Practices Using Artificial Intelligence (AI): A Survey", the rows represent responses ("Yes" and "No") and the columns represent different survey questions:

[1] Awareness of artificial intelligence in dentistry

[2] Implementation of artificial intelligence tools

[3] Use of artificial intelligence in diagnostics

[4] Belief that artificial intelligence will become a standard in future dental practices

## Key findings:

[1] The majority of participants are aware of artificial intelligence (120 out of 150).

[2] 90 participants have already implemented artificial intelligence in their dental practice.

[3] 45 participants primarily use artificial intelligence for diagnostics.

[4] A large number of participants (130) believe artificial intelligence will be the future of dental practice.

## Discussion:

The findings of this study underscore a significant and growing trend in the adoption of Artificial Intelligence (AI) within dental practices, with diagnostics emerging as the most frequently reported application. The survey highlights that while a substantial number of dental professionals are aware of artificial intelligence technologies and have implemented them-particularly in diagnostic tools-there remains a notable portion who have not yet adopted these technologies. This discrepancy suggests that barriers such as cost, training and knowledge gaps may be influencing the rate of artificial intelligence integration [12]. Cost remains a primary obstacle for many dental practices, as artificial intelligence tools and technologies can require substantial investment [13]. Smaller or independent practices may find it challenging to allocate the necessary funds for these advanced systems. Training is another critical factor; effective utilization of artificial intelligence technologies requires dental professionals to acquire new skills and knowledge, which can be time-consuming and may involve additional expenses for training programs [14]. Furthermore, a lack of knowledge about the benefits and functionalities of artificial intelligence tools could deter some practitioners from adopting these innovations, as they may not fully understand how artificial intelligence can enhance their practice [15, 16].

Despite these barriers, the majority of participants in the study agreed on the positive impact of AI, particularly in enhancing treatment outcomes [17, 18, 19- 20]. Many believe that artificial intelligence has the potential to significantly improve diagnostic accuracy, streamline treatment planning and optimize patient management, leading to better overall care [21, 22-23]. Eschert *et al.* in a study of 302 dentists found that 37.1% rated their artificial intelligence knowledge as average, while concerns included machine errors (3.7 ± 1.3) and data security (3.5 ± 1.24) [22]. This optimism is reflected in the high percentage of respondents who anticipate artificial intelligence becoming a standard component of future dental practices [24, 25]. Ayad *et al.* this study of 265 patients found that key concerns about artificial intelligence in dentistry included workforce impact (37.7%) and doctor-patient relationships (36.2%), while major expected benefits were improved diagnostics (60.8%) and time efficiency (48.3%). Most patients anticipated artificial intelligence integration within 1-10 years, with older patients (>35 years) expecting higher performance standards (p < 0.05). Understanding patient perceptions can help shape the future of AI-driven dentistry [26]. Artificial intelligence has the potential to revolutionize dentistry by enhancing diagnostics in periodontal disease and cariology through advanced models like artificial neural network and convolutional neural network. This review explores AI's role in detecting early decay, analyzing bone loss and improving treatment precision in various dental specialties. However, widespread artificial intelligence adoption in dentistry faces challenges that must be addressed for successful integration [27]. Artificial intelligence in endodontics aids in root canal anatomy analysis, pulp stem cell viability prediction, working length measurement, lesion detection and retreatment outcome forecasting using models like convolutional neural network and artificial neural network [28]. Artificial intelligence (AI) has been integrated into all dental disciplines, including operative dentistry, periodontics, orthodontics, oral and maxillofacial surgery and prosthodontics. While artificial intelligence is primarily utilized for diagnosis through radiographic and optical image analysis, its application in other areas remains limited due to challenges in data availability, uniformity and computational requirements for 3D data processing. Evidence-based dentistry remains the gold standard for clinical decision-making, while artificial intelligence machine learning models, trained on human expertise, serve as valuable tools to support dental professionals across various stages of patient care [29]. Hegde *et al.* highlights the evolving role of artificial intelligence in dentistry, with a majority of Australian dentists and dental students recognizing its potential as a supportive tool. While awareness of artificial intelligence applications is relatively high, specific knowledge about existing artificial intelligence software remains limited. The findings suggest optimism regarding AI's integration into clinical practice, particularly among younger professionals and students, though concerns persist regarding job security, adaptability in patient care and trust in artificial intelligence accuracy. Understanding these perceptions is crucial for developing AI-driven solutions that align with the expectations and needs of dental practitioners [30]. Artificial intelligence and neural networks enhance efficiency, accuracy and time-saving in dentistry, though further research is needed for routine clinical integration [31]. Artificial intelligence enhances precision and predicts clinical failures in dentistry, yet its full potential requires further research and validation [32]. Artificial intelligence models achieve high accuracy (93.8%-98%) in implant type recognition using dental images. Prediction models for implant success show moderate accuracy (62.4%-80.5%), while AI-driven implant design optimization enhances biomechanical performance. Despite promising results, further development and validation are needed [33]. Innovative inter-professional collaboration among clinicians, researchers and engineers is crucial for advancing artificial intelligence in dentistry. Despite concerns about misinterpretation and patient privacy, artificial intelligence integration will enhance precision in treatment and facilitate real-time information exchange. This progress will enable data-driven insights, improving patient care across healthcare systems [34]. The study suggests that to overcome the barriers to artificial intelligence adoption, efforts should be focused on addressing cost concerns through financial support or scalable solutions, improving training programs to ensure practitioners can effectively use artificial intelligence tools and increasing awareness about the benefits of artificial intelligence in dentistry. As artificial intelligence technology continues to evolve and become more accessible, it is likely that its integration into dental practices will become more widespread, ultimately leading to enhanced patient care and more efficient practice management.

## Conclusion:

The integration of artificial intelligence into dental practice is steadily increasing, particularly in diagnostic and treatment planning applications. With high awareness and positive reception, it is expected that artificial intelligence will play a significant role in shaping the future of dentistry.

## Figures and Tables

**Table 1:** Data overview

**Question**	**Responses (n=150)**	**Percentage (%)**
Are you aware of artificial intelligence technologies in dentistry?	Yes (120), No (30)	80%, 20%
Have you implemented artificial intelligence tools in your practice?	Yes (90), No (60)	60%, 40%
Primary application of artificial intelligence in your practice?	Diagnostics (45), Treatment Planning (30), Patient Management (15)	50%, 33%, 17%
How effective do you find artificial intelligence for improving patient outcomes?	Highly effective (45), Moderately effective (75), Not effective (30)	30%, 50%, 20%
Do you believe artificial intelligence will become a standard in dental practice?	Yes (130), No (20)	87%, 13%
**Question**	**Responses (n=150)**	**Percentage (%)**
1. What is your primary dental specialization?	General Dentistry	40%
	Orthodontics	20%
	Endodontics	15%
	Periodontics	10%
	Prosthodontics	10%
	Oral Surgery	5%
	Other	0%
2. How many years have you been practicing Dentistry?	Less than 5 years	25%
	5-10 years	35%
	11-20 years	25%
	More than 20 years	15%
3. What type of dental practice do you work in?	Private Practice	45%
	Group Practice	25%
	Hospital Setting	15%
	Academic Institution	10%
	Other	5%
4. Are you aware of artificial intelligence technologies being used In dentistry?	Yes	80%
	No	20%
5. Have you received any formal training on ARTIFICIAL INTELLIGENCE tools in dental practice?	Yes	35%
	No	65%
6. Do you use artificial intelligence technologies in your practice?	Yes	60%
	No	40%
7. If yes, in which area do you use artificial intelligence the most?	Diagnostics	50%
	Treatment Planning	25%
	Patient Management	15%
	Predictive Analytics	10%
8. What type of artificial intelligence tools do you use in your practice?	AI-powered Diagnostic Software	55%
	Virtual Assistants	20%
	AI-based Treatment Planning Systems	15%
	AI-based Telemedicine Platforms	5%
	Other	5%
9. How effective do you believe artificial intelligence is in improving Diagnostic accuracy?	Highly Effective	60%
	Moderately Effective	30%
	Not Effective	10%
10. How effective do you find artificial intelligence in enhancing Treatment outcomes for patients?	Highly Effective	50%
	Moderately Effective	35%
	Not Effective	15%
11. Do you think artificial intelligence has reduced the overall time Spent on patient management and administrative tasks?	Yes	55%
	No	45%
12. What are the biggest barriers to artificial intelligence adoption in Dental practices?	Cost of artificial intelligence Tools	40%
	Lack of Training	30%
	Data Privacy Concerns	15%
	Integration with Existing Systems	10%
	Resistance to Change	5%
13. Do you believe artificial intelligence will become a standard in dental Practice in the next 5-10 years?	Yes	85%
	No	15%
14. What artificial intelligence technologies do you expect to see more Widely used in the future?	AI-powered Diagnostic Systems	50%
	AI-based Predictive Models for Treatment Outcomes	30%
	ARTIFICIAL INTELLIGENCE in Patient Education and Communication	10%
	ARTIFICIAL INTELLIGENCE in Robotic-assisted Surgeries	5%
	Other	5%
15. Would you be willing to adopt more artificial intelligence tools if cost And training was not an issue?	Yes	75%
	No	25%
